# Induction of VMAT-1 and TPH-1 Expression Induces Vesicular Accumulation of Serotonin and Protects Cells and Tissue from Cooling/Rewarming Injury

**DOI:** 10.1371/journal.pone.0030400

**Published:** 2012-01-12

**Authors:** Fatemeh Talaei, Martina Schmidt, Robert H. Henning

**Affiliations:** 1 Department of Clinical Pharmacology, University Medical Center Groningen, University of Groningen, Groningen, The Netherlands; 2 Department of Molecular Pharmacology, Faculty of Pharmacy, University of Groningen, Groningen, The Netherlands; National Institutes of Health, United States of America

## Abstract

DDT_1_ MF-2 hamster ductus deferens cells are resistant to hypothermia due to serotonin secretion from secretory vesicles and subsequent cystathionine beta synthase (CBS) mediated formation of H_2_S. We investigated whether the mechanism promoting resistance to hypothermia may be translationally induced in cells vulnerable to cold storage. Thus, VMAT-1 (vesicular monoamino transferase) and TPH-1 (tryptophan hydroxylase) were co-transfected in rat aortic smooth muscle cells (SMAC) and kidney tissue to create a serotonin-vesicular phenotype (named VTSMAC and VTkidney, respectively). Effects on hypothermic damage were assessed. VTSMAC showed a vesicular phenotype and an 8-fold increase in serotonin content and 5-fold increase in its release upon cooling. Cooled VTSMAC produced up to 10 fold higher concentrations of H_2_S, and were protected from hypothermia, as shown by a 50% reduction of caspase 3/7 activity and 4 times higher survival compared to SMAC. Hypothermic resistance was abolished by the inhibition of CBS activity or blockade of serotonin re-uptake. In VTkidney slices, expression of CBS was 3 fold increased in cold preserved kidney tissue, with two-fold increase in H_2_S concentration. While cooling induced substantial damage to empty vector transfected kidney as shown by caspase 3/7 activity and loss of FABP1, VTkidney was fully protected and comparable to non-cooled control. Thus, transfection of VMAT-1 and TPH-1 induced vesicular storage of serotonin which is triggered release upon cooling and has protective effects against hypothermia. The vesicular serotonergic phenotype protects against hypothermic damage through re-uptake of serotonin inducing CBS mediated H_2_S production both in cells and kidney slices.

## Introduction

Hypothermia is potentially harmful to cell viability [1]. DDT_1_ MF-2 cells (DDT_1_), which are ductus deferens smooth muscle cells derived from Syrian golden hamster (*Mesocricetus auratus*), a natural hibernating animal, are resistant to hypothermia and do not show cell death following hypothermia and rewarming of cells *in vitro* [2]. We previously found this hypothermic resistance of DDT_1_ cells to be conveyed by secretion of serotonin from secretory vesicles in DDT_1_, and subsequent re-uptake inducing the formation of H_2_S through cystathionine beta synthase activation [2]. One of the effects of hypothermia is membrane depolarization [3], which would stimulate secretion by secretory cells [4] due to the activation of voltage-dependent calcium channels, leading to cell contraction [5]. The storing vesicles in secretory cells contain different substances including collagen, lipids and neurotransmitters, which are liberated into the medium during secretion [6].

In contrast to DDT_1_ cells, rat smooth muscle aorta cells (SMAC) are highly vulnerable to hypothermic damage [2]. Many cell types are vulnerable to hypothermia mainly due to the burst of reactive oxygen species (ROS) during the re-warming phase leading to low ATP production, Ca^2+^ overload and cell swelling, resulting in apoptotic cell death [7], [8]. Oxidative damage is both present during ischemia/reperfusion and hypothermia/rewarming [9]. In addition to its stimulation of H_2_S production, serotonin attenuates ROS induced damage through scavenging of ROS [10]. Thus, we hypothesized that the induction of a serotonergic vesicular phenotype in SMAC would induce resistance to hypothermia/rewarming damage and cell death by the activation of a secretory system from vesicular structures present in cells and subsequent production of H_2_S.

In our previous study in cells from hibernating animals [2], we found that cooling induces the release of serotonin. Whether cells that store dopamine also release this compound upon cooling, is still unknown. Thus, we chose to induce a vesicular phenotype containing serotonin. Accordingly, the sequences for TPH-1 (tryptophan hydroxylase-1) and VMAT-1 (vesicular monoamine transporter-1) were transfected into SMAC and kidney tissue slices. Different properties in relation to morphology, protein induction, and cell survival were analyzed after hypothermic preservation and rewarming. Here we demonstrate that the introduction of TPH-1 and VMAT-1, representing key components of the vesicular serotonergic pathway, leads to hypothermia induced secretion of serotonin in vitro in SMAC. Further, we demonstrate that the induction of this vesicular phenotype protects the integrity of SMAC and of kidney tissue slices in hypothermia/rewarming damage through the H_2_S pathway.

## Materials and Methods

### Cell culture

DDT_1_ MF2 (hamster ductus deferens muscle cells, ATCC, USA CRL1701) cultured in DMEM (Gibco) and SMAC (rat smooth muscle aortic cells, ATCC, USA CRL1476) cultured in DMEM/F12 (Gibco, Belgium) were chosen for this study. The media was supplied with 10% (v/v%) fetal calf serum and 100 U/ml penicillin, 100 µg/ml streptomycin and cells were cultured at 37°C in 5% CO_2_ in 25 cm^2^ or 75 cm^2^ flasks. Morphology of the cells was performed by phase contrast light microscopic examination. To study the necessity of the presence of serotonin and CBS in H_2_S production and hypothermia resistance, cells were treated with vehicle, amino oxyacetic acid (AOAA, 10 mM), an inhibitor of H_2_S production, or fluoxetine (1 µM), an inhibitor of the serotonin reuptake. Culture medium was collected after 24h of hypothermic treatment (3°C).

### Quantification of collagen

Picrosirius red was used to quantify the amount of collagen in cells and the supernatant of cells. To prepare Picrosirius red solution, 0.2 g Sirius Red F3B was added to 200 ml of saturated aqueous solution of picric acid. Cells were switched to 5% FBS medium prior to experiments. Cells at 37°C and at 3°C (18h) were lysed in RIPA buffer. Duplicate 50 to 100 µl aliquots were added to 1 ml of dye solution and mixed at room temperature for 30 min, and centrifuged at 10,000 g for 5 minutes to pellet the collagen. One ml of 0.1 M HCl was added to each tube to remove unbound dye flowed by centrifugation at 10,000 g for 5 min. One ml of 0.5 M NaOH was added and vortexed vigorously to release the bound dye. Absorption was read at 540 nm. Collagen stock solution (2 mg/ml) was prepared by adding 5 ml of 0.5 M sterile acetic acid to 10 mg type I Collagen lyophilized powder (Sigma C-3511). A collagen standard curve was obtained using 12.5 to 75 µg of collagen [11].

### Quantification of serotonin

Ehrlich's reagent was used to quantify the cellular amount of indoles [12]. Five ml of medium was shaken vigorously with 2 ml of xylene. Next, 1 ml of Ehrlich's reagent was applied to the surface of the mixture. Redistribution of xylene through the Ehrlich's reagent induces formation of the rosindole body, indicating the presence of indoleamides. To measure the intracellular indole concentration, cells were washed with PBS, centrifuged (1000 rpm, 5 min) and the supernatant was removed. Four ml of ethyl acetate was used to extract the serotonin from the supernatant to achieve a dry extract. Ehrlich reagent (200 µl) was added to either the cell pellet or the extract. After 3 min of vortexing, tubes were left for 3h at 60°C and centrifuged (1000 rpm, 5 min). Indole content was quantified spectrophotometrically at 625 nm. Calibration experiments were carried out using serotonin (0.025–0.5 mM), which rendered a linear regression with a correlation coefficient (R^2^) of 0.9996 (data not shown).

### Constructs

RNA was isolated from brain of male Sprague-Dawley rats using a nucleospin RNA II kit (Macherey-Nagel, UK) and used for cDNA synthesis. Sequences were amplified by PCR using primers designed to produce the full length coding regions. Rat TPH-1 cDNA (1.564 bp) was obtained by amplifying 25 ng of cDNA using GAACTCCAGTGGCTTTGAGG (forward) and CAGAGAGGTGAGAGACATTGCT (reverse) [13]; and for VMAT-1 (1.831 bp) by using 5′-CCAGGCAGACTTCTTCTCCTATAAA-3′ (forward) and 5′-GCACTTACAGGTGAGTAAAGGAAAGGTA-3′ (reverse) [14]. The PCR products were purified by Nucleospin Extract II (Cat NO. 740609.50, Machery-Nagel, Netherlands) and size was checked by electrophoresis on a 1.5% TAE agarose gel at 100V for 20 min (BIO-RAD, PowerPac 300). The sequences were inserted into pTARGET (Promega**,** A1410) and transformed into 5 HD-α E. coli. Plasmid DNA from positive colonies were isolated (nucleobond PC100, Machery-Nagel, Netherlands). Correct insertion of the gene was checked by digestion with EcoRI (Promega, US). The concentration of plasmid DNA was measured using a NanoDrop spectrophotometer (Life Science ND1000, US).

### SMAC transfection with TPH-1 and VMAT-1 plasmids and protein expression analysis

SMAC were plated in 6 well plates at 50% confluence in DMEM/F12 medium free of antibiotic and FCS (Gibco, E12-719F). Plasmids containing the sequences were introduced into the cells using lipofectamine 2000 according to the manufacturer's protocol (Invitrogen, UK). After 24h at 37°C, the supernatant was replaced with FBS containing normal cell medium and the cells were left to proliferate for another 24h at 37°C. The double transfected cells are referred to as VTSMAC (VMAT-1/TPH-1 smooth muscles aortic cells.) Control cells were transfected with the empty pTARGET vector following the same procedure.

Western blot analysis was performed to investigate the expression of VMAT-1 and TPH-1 in cells. Cell samples were homogenized in ice-cold RIPA buffer [15]. Twenty µl of loading buffer (10% SDS, 50% Glycerol, 0.33 M Tris HCl pH 6.8, 0.05% bromophenol blue) was added to 50 µg of protein and loaded onto pre-made gels 4–20% (Thermoscientific 15 wells #25224) for electrophoresis at 100 V (80 min) and blotting on a nitrocellulose membrane. VMAT-1 (santa cruz, SC15313) and TPH-1 (santa cruz, SC30079) specific antibodies (1∶1000, overnight at 3°C) were used to detect these proteins. The membranes were washed three times with TBS+Tween solution and treated with the related secondary antibodies (1∶1000, 2h at room temperature). The membranes were developed using super signal West Dura substrate. Genesnap (Syngene version 6.07) was used to acquire images and results were analyzed using Genetools version 3.08.

### Cell staining and H_2_S production

For immunohistochemical examination, cells were fixed by acetone (100%) for 10 min, washed and rehydrated with PBS. Hydrogen peroxidase activity was blocked by hydrogen peroxide (1%) in PBS for 30 min, washed with PBS three times for 5 min each and incubated for 1 h with 1% primary antibody to serotonin (Abcam, ab8882-50) in PBS containing 1% BSA for 1 h, washed in PBS thrice and incubated with 1% secondary antibody Goat AntiRabbit HRP (Dako, po448) in PBS containing 1% BSA for 1 h. The signal was amplified by 1% of a third antibody, rabbit Anti Goat (Dako, po449) in medium. The slides were washed in PBS and Dako AEC+High sensitivity substrate chromogen (Dako, Denmark) was used to visualize the stain. Hematoxyline counterstaining was performed to visualize the cell nuclei. For examination of collagen, cells were stained with saturated saffron (Sigma) solution in 0.07 M borate buffer (pH 10) for 30 min at room temperature. Staining of VMAT-1 and CBS was performed using antibodies to VMAT-1 and CBS (santa cruz, sc-46830) in PBS containing 1% BSA, similar to the procedure with secondary and tertiary antibodies as described above. The methylene blue method was used to measure H_2_S production in 4 ml of cell free supernatant. Zinc acetate (1%) was added to each sample to trap the H_2_S in the supernatant. Diamine-ferric solution was prepared by mixing 100 µl of a 400 mg N,N-dimethyl-p-phenylenediamine dihydrochloride dissolved in 10 ml 6M HCl and 100 µl of 600 mg ferric chloride in 10 ml 6M HCl. Two hundred µl of this mixture was added to the sample, incubated for 30 min at 37°C. The amount of methylene blue was measured at 670 nm [16], [17]. The concentration of H_2_S was calculated using a methylene blue standard curve.

#### siRNA for cystathionine-β-synthase in VTSMAC

The expression of CBS in VTSMAC was reduced by applying a predesigned siRNA (sc-60336, Santa Cruz) and compared to a silencer negative control (Ambion, AM4644). VTSMAC cells at 60–80% confluence were seeded in 96 or 6 well plates in antibiotic-free normal growth medium supplemented with FCS. Cells were transfected using lipofectamine 2000 (Invitrogen) at a final concentration of 100 pmol siRNA in 5 µl lipofectamine for each well in a 6 well plate and 5 pmol siRNA in 0.25 µl lipofectamine for each well in a 96 well plate. After 24h, the medium was changed to the medium containing antibiotics and FCS. Cells were left to proliferate for 48h at 37°C. Then, control VTSMAC, siRNA treated VTSMAC and VTSMAC transfected with the negative control silencer were incubated at 37°C or 3°C. Cell viability was measured by MTS assay (Promega). 20 µl of MTS solution was added to each well and cells were subsequently placed at 37°C in 5% CO_2_ for 3h. Absorption was read at 490 nm.

### Caspase 3/7 activity and MTS assay

Cells were cultured in a 96 well plate and placed at 37°C for 48h till confluence. The cells were treated for 15 min with vehicle, fluoxetine (1 µM) or AOAA (10 mM) and then placed at 3°C for 24h. Caspase 3/7 activity was measured by adding 50 µl of Apo-ONE® Homogeneous Caspase-3/7 reagent (G7792, Promega, Germany) after shaking for 3h. Fluorescence was read at an excitation of 499 nm and emission of 521 nm. Cell viability was measured by MTS assay.

### Kidney slice transfection with TPH-1 and VMAT-1 plasmids

Kidneys were surgically removed from two male Sprague-Dawley rats (450 g) after euthanization under isoflurane anesthesia, flushed with ice cold PBS and cut into 3 millimeter thick sections. Slices immediately deep frozen or fixed in a zinc fixative solution (0.1 M Tris-HCl pH 7.8, 0.05% calcium acetate, 0.5% zinc acetate, 0.5% zinc chloride) served as 37°C control. The TPH-1 and VMAT-1 or empty vectors were transfected into the remaining slices using Lipofectamine-2000 (Invitrogen) at 10 ng/µl for each vector in 300 µl of DMEM-MF12 medium without FBS at 37°C for 18 h. Then, medium was changed to DMEM supplemented with 10% FCS and 100 U/ml penicillin, 100 µg/ml streptomycin. Thereupon, the tubes were transferred to 3°C for 24h following 1 hr of rewarming at 37°C. The animal experiment was approved by the Animal Experiments Committee of the University of Groningen, The Netherlands (DEC#5920).

### Tissue damage in kidney after tranfection and NaHS treatment

We have previously shown that NaHS protects SMAC against hypothermia rewarming injury [2] to further compare the protective effects of TPH-1 and VMAT-1 transfection to the direct effects of NaHS addition to kidney medium. For this purpose, untreated kidney slices that were cooled for 24h at 3°C were treated with sodium hydrosulfide (NaHS, 0.3 mM) immediately before rewarming, to assure the presence of H_2_S during rewarming. Non-treated kidney slices served as controls. Caspase 3/7 activity, western blotting and immunohistochemistry were used. Caspase activity was measured in 500 µg of protein from tissue lysed in 50 mM potassium phosphate buffer (pH 6.9, 5%, w/v), sodium orthovanadate 10 mM and protease inhibitor (50 µl). Fifty µl of substrate (Promega Apo-ONE®) was added and shaken for 3h.

Expression of Fatty Acid Binding Protein1 (FABP1) was used to detect ischemic kidney injury [18]. Kidney samples were homogenized in ice-cold RIPA buffer. 50 µg of protein was loaded onto pre-made gels and the process of western blotting was followed as previously described. FABP1 was detected using a specific antibody (1∶1000; Santa Cruz, SC50380) followed by a secondary antibody. The bands were detected as previously described for VMAT-1 and TPH-1.

For immunohistochemistry, samples were fixed and embedded in paraffin, cut in 3 µm sections, deparaffinized and submitted to antibody staining for VMAT-1, CBS, and FABP1. Sections were incubated with primary antibodies (1∶100) with 1% BSA for 1 hr and subsequently washed three times with PBS. Next, sections were incubated with secondary antibodies (1∶100) with 1% BSA and 1% rat serum for an hour and subsequently washed 3 times with PBS. A fluorescent second antibody (1∶100) with 1% BSA and 1% rat serum was added against FABP1 antibody. For all the stains except FABP1 Dako AEC was used to visualize the primary antibody and hematoxyline counterstaining was performed to visualize the cell nuclei. Dako fluorescence mounting medium+DAPI was used to visualize FABP1 stains.

### H_2_S production in kidney

Pieces of kidney were homogenized in ice-cold 50 mM potassium phosphate buffer, pH 8.0 (12% wt/vol) on dry ice. The homogenate was centrifuged (47,000 *g*; 10 minutes; 4°C) and the supernatant (75 µl), mixed with 0.25 ml Zn acetate (1%) and 0.45 ml water for 10 minutes at room temperature. TCA (10%; 0.25 ml) was then added and centrifuged (14.000 *g*; 10 minutes; 4°C). The clear supernatant was mixed with *N,N-*dimethyl-p-phenylenediamine sulfate (20 mM; 133 µl) in 7.2 M HCl and FeCl_3_ (30 mM; 133 µl) in 1.2 M HCl. After 20 minutes, absorbance was measured at 670 nm. Blanks were made following the same procedure without samples. The concentration of H_2_S was calculated as outlined above.

### Statistics

Data are presented as mean ± SEM. Statistical data analyses were performed using a One-way ANOVA (P<0.05) with Tukey post-hoc testing (GraphPad Prism version 5.00 for Windows, GraphPad Software, San Diego California USA), unless indicated otherwise.

## Results

### VTSMAC microscopy

**Figure 1:**
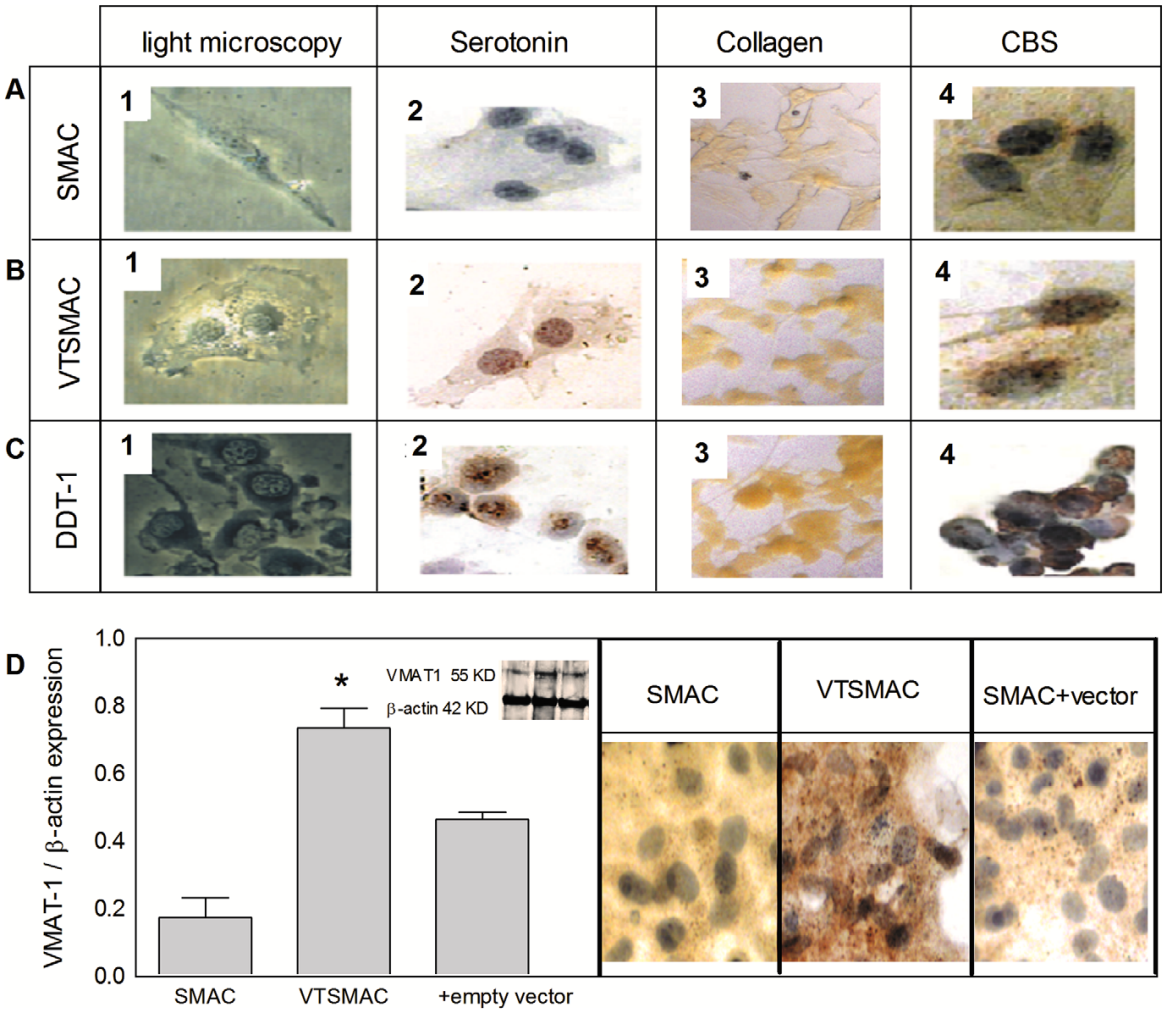
Co-expression of rat tryptophan hydroxylase-1 (TPH-1) and vesicular monoamine transporter-1 (VMAT-1) in smooth muscle aorta cells (SMAC) induces a serotonergic vesicular phenotype. Effects are compared between aortic cells transfected with empty vector (SMAC), with vectors containing coding sequences (VTSMAC) and natural resistant DDT_1_ cells. A to C, microscopic evaluation demonstrating the presence of vesicles (subpanels 1), antibody staining reveals the presence of mainly nuclear serotonin (subpanels 2), collagen (subpanels 3) and CBS (subpanels 4) in VTSMAC and DDT_1_, whereas expression is absent or lower in SMAC. Original magnification 40x. D, VMAT-1 expression is increased in VTSMAC compared to SMAC. The data are Mean ± SEM (n = 3); p<0.005; ANOVA.

SMAC were studied at 48h after transfection with both TPH-1 and VMAT-1 or empty vector. The transfected SMAC were named VTSMAC (Fig.1 B). VTSMAC demonstrated a higher number of cellular vesicles (Fig.1 B1) compared to SMAC (Fig.1 A1), very similar to those found in DDT_1_ (Fig.1 C1). Serotonin antibody stain showed the presence of serotonin as red dots in VTSMAC, primarily localized at the nucleus (Fig.1 B2), which was similar to DDT-1 (Fig.1 C2), while SMAC lacked their presence (Fig.1 A2). Transfection with TPH-1 and VMAT-1 yielded a typical transfection efficiency of 65% as measured by serotonin antibody staining and counting of cells that expressed intracellular serotonin after transfection. The expression of VMAT-1 and TPH-1 proteins after transfection were measured using western blot analysis. VTSMAC displayed a significant upregulation of VMAT-1 protein levels compared to control SMAC and empty vector transfected cells (Fig.1 D). Similar results were observed for TPH-1 protein (Fig. S1A).

Saffron dye staining showed the presence of collagen as bright yellowish coloring in VTSMAC (Fig.1 B3), with higher intensity compared to SMAC (Fig.1 A3), and at similar levels as found in DDT_1_ (Fig.1 C3). CBS antibody stain demonstrated the presence of this protein inside the VTSMAC. CBS protein was localized in cytoplasmic and nuclear compartments (Fig.1 B4). CBS protein was also observed in SMAC (Fig.1 A4) and DDT_1_ (Fig.1 C4).

### Serotonin and collagen concentration

**Figure 2:**
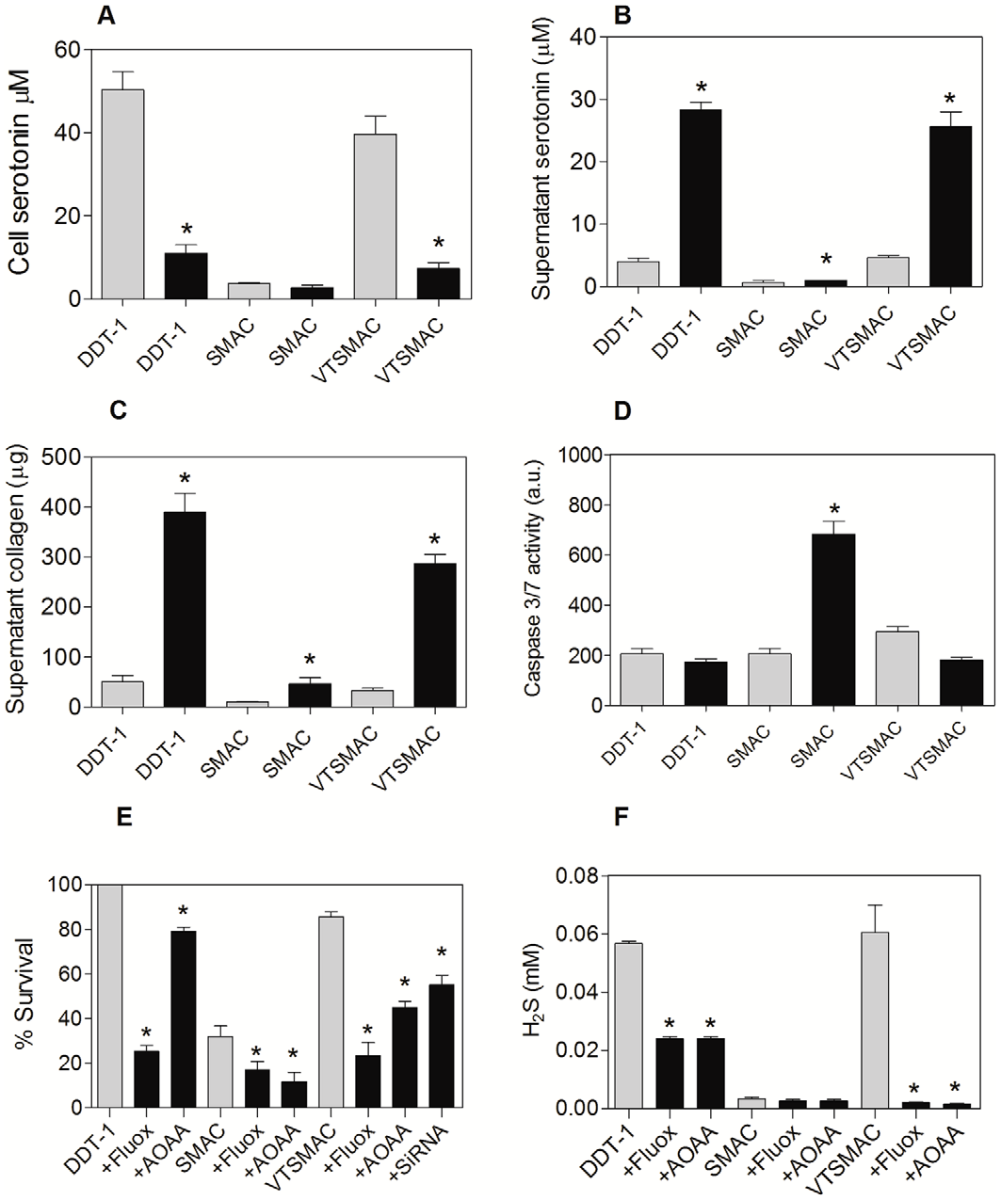
The serotonin vesicular phenotype protects VTSMAC from hypothermic damage through serotonin reuptake and CBS mediated production of H_2_S. A, cellular levels of serotonin are increased in VTSMAC compared to SMAC at 37°C and decrease upon cooling. B, Upon cooling, serotonin is released from VTSMAC and DDT_1_ as demonstrated by increased levels of serotonin in the supernatant. C, release of collagen in the supernatant upon cooling in VTSMAC and DDT_1_. D, caspase 3/7 activity is normalized in VTSMAC compared to SMAC following hypothermia. E, MTS assay shows increased survival of VTSMAC after 24h at 3°C compared to control SMAC. The augmentation of cell survival in VTSMAC is abrogated by treatment with AOAA (10 mM), fluoxetine (1 µM) or SiRNA against CBS. F, H_2_S content of supernatant of cells following cooling (24h, 3°C). VTSMAC show increased H_2_S levels compared to SMAC, which is inhibited by AOAA and fluoxetine. Gray bars represent controls in each cell line (37°C) and black bars represent cells after static hypothermic storage (3°C, 24h). * represent significant difference to control bars in each group. The data are Mean ± SEM (n = 3); p<0.005; ANOVA.

The intracellular concentration of serotonin in VTSMAC was 5 times increased compared to SMAC, and ranged up to 2/3 of the concentration measured in DDT_1_ cells (Fig. 2A). Similarly to DDT_1_ cells, the concentration of serotonin in the supernatant of the VTSMAC after 18h of hypothermia treatment at 3°C increased 6 fold, while no increase was found in supernatant of cooled SMAC (Fig. 2B). The concentration of collagen in the supernatant of the VTSMAC after 18h of hypothermia treatment at 3°C increased 5 fold (Fig. 2C). While a similar increase was observed in hypothermic DDT_1_ cells, cooling of SMAC did not induce an increase of the collagen concentration of supernatant (Fig. 2C). Collectively, the above data demonstrate that VTSMAC display a serotonin vesicular phenotype.

### Hypothermia resistance of VTSMAC involves serotonin and H_2_S

Caspase 3/7 activity in cells was investigated as an apoptotic marker before and after hypothermia treatment of VTSMAC, SMAC and DDT_1_ (Fig. 2D) in cells cooled for 24h and re-warmed for 3h. Cooled VTSMAC had similar caspase activity compared to 37°C control cells, while cooled SMAC showed a significant increase in caspase 3/7 activity (Fig. 2D). The viability of VTSMAC was verified by MTS assay after incubation of the cells for 24h at 3°C and rewarming for 3h. Whereas 80% of VTSMAC survived the cooling/rewarming compared to non-cooled VTSMAC, survival in untransfected SMAC amounted only 30% (Fig. 2E). Further, DDT_1_ cells showed full survival of cooling and ewarming. CBS SiRNA almost completely downregulated CBS expression (Fig. S1). Cell survival in VTSMAC was decreased by treatment with fluoxetine and AOAA (aminooxy acetic acid), an inhibitor of CBS activity and by siRNA against CBS. DDT1 cell survival was mainly affected by fluoxetine (Fig. 2E).

H_2_S has been shown to exert protective effects in hypothermic damage of SMAC. To verify the hypothesis that VTSMAC show resistance to cooling because of H_2_S production, its concentration was measured in the absence and presence of fluoxetine or AOAA. Cooling at 3°C for 18h induced a 7–10 fold increase in the H_2_S concentration present in the supernatant of VTSMAC, while its concentration in SMAC was unchanged (Fig. 2F). Both fluoxetine and AOAA drastically reduced the content of H_2_S in the supernatant of all cells. DDT_1_ showed less inhibition of H_2_S production by either substance compared to both SMAC and VTSMAC (Fig. 2F).

Taken together, these data imply that in VTSMAC, re-uptake of serotonin results in the production of H_2_S through CBS, in turn protecting cells against hypothermia/rewarming damage.

### Kidney transfection with TPH-1 and VMAT-1 attenuates hypothermic damage

**Figure 3:**
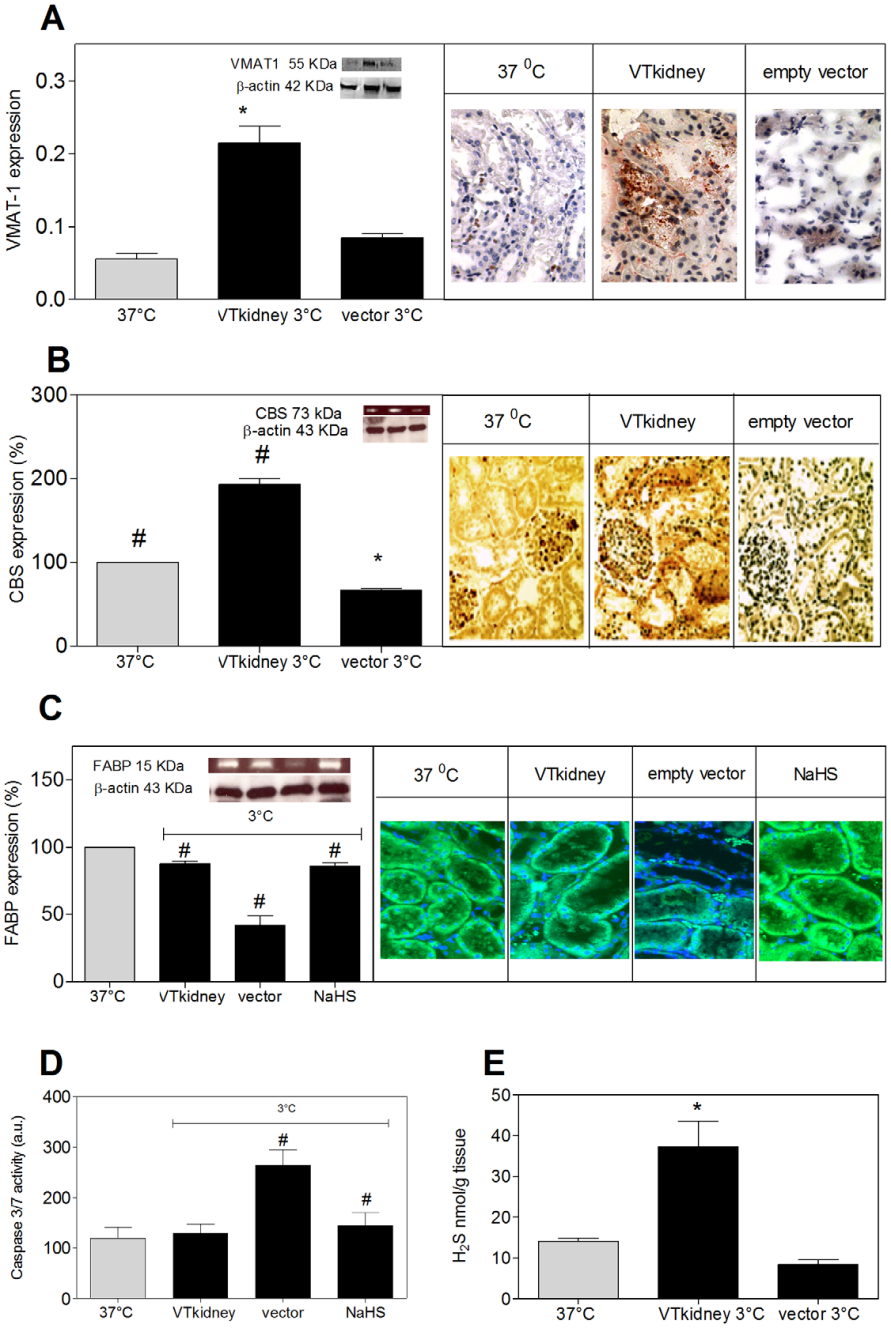
Co-expression of rat vesicular monoamine transporter-1 (VMAT-1) and tryptophan hydroxylase-1 (TPH-1) in kidney slices (VTkidney) and treatment of kidney slices with NaHS (0.3 mM) protect tissue against static hypothermic storage (3°C, 24h) by induction of cystathionine beta synthase (CBS) and H_2_S production. A, VTkidney show increased expression of VMAT-1. B, VTkidney shows increased expression of CBS, with main presence in tubular cells. C, VTkidney and NaHS treated slices show absence of hypothermia induced loss of fatty acid binding protein (FABP1). FABP1 staining (green) was predominantly in the cytoplasmic region of the proximal tubule. D, VTkidney and NaHS treated slices lack hypothermia induced increased expression of caspase 3/7 activity. E, VTkidney shows increased production of H_2_S. Data are means ± SEM. * denotes difference from non-cooled tissue (Controls 37°C), # denotes difference from empty vector hypothermic tissue (3°C), ANOVA tests, p<0.05. Gray bars represent controls and black bars hypothermia treated tissue. Experiments consist of n ≥3. All magnifications are 40x.

To assess the potential of VMAT-1 and TPH-1 transfection in protecting kidney tissue against hypothermia/rewarming damage, kidney slices were transfected with TPH-1 and VMAT-1 (VTkidney) or empty vector. The success of the transfection was assessed through VMAT-1 antibody staining and immunoblot analysis. The intensity of the VMAT-1 stain increased in kidneys transfected with VMAT-1 and TPH-1 compared to empty vector, mainly in tubular cells (Fig. 3A). Immunoblotting showed a doubling of the expression of VMAT-1 (Fig. 3A) and TPH-1 (Fig. S1B) in VTkidney compared to control tissue.

Hypothermic storage downregulated FABP1 expression by 50% (Fig. 3C) and doubled activity of caspase 3/7 (Fig. 3D) in empty vector treated tissue. In contrast, in VTkidney and NaHS treated kidney slices, caspase activity and FABP1 expression was unaffected by hypothermic storage and maintained at control levels.

To substantiate involvement of the H_2_S system, CBS expression in kidney slices was measured by western blotting and immunohistology. In VT kidney following hypothermic storage, CBS expression was doubled compared to empty vector transfected tissues, which show lower expression of CBS compared to 37°C controls (Fig. 3B). The same was observed in immunostaining, in which CBS was mainly present in the endothelium and the epithelium of kidney glomeruli and in the tubuli (Fig. 3B). Finally, H_2_S concentration was more than doubled in transfected tissue compared to controls (Fig. 3E).

Thus, VTkidney slices show an increase in the expression of VMAT-1 and CBS, increased production of H_2_S, and attenuation of renal damage following hypothermia/rewarming, as evidenced by maintained FABP1 staining and normalized caspase 3/7 activity.

## Discussion

This is the first account of cells becoming resistant to hypothermia rewarming damage after the induction of a vesicular serotonergic phenotype, as accomplished by transfection with TPH-1 and VMAT-1. Induction of the serotonergic vesicular phenotype was shown by increased staining for serotonin and increased expression of VMAT-1 and TPH-1. Resistance to hypothermia (3°C) and rewarming (37°C) in transfected smooth muscle aorta cells, named VTSMAC, was demonstrated by reduced apoptosis and increased viability of cells. Further, as shown previously in DDT_1_ cells, which show natural resistance to hypothermia [2] the resistance of VTSMAC to hypothermia was also mediated by CBS dependent production of H_2_S following the induction of the vesicular serotonergic phenotype. In addition, transfection of kidney slices with vectors containing TPH-1 and VMAT-1 sequences increases their viability after hypothermic storage, as demonstrated by a normal level of caspase 3/7 activity and FABP1 expression. Further, kidney tissue transfected with plasmids containing TPH-1 and VMAT-1 sequences showed increased production of H_2_S, which is in accordance with the observed increase in CBS expression. NaHS treatment of kidney slices showed similar results as transfection with TPH-1 and VMAT-1, with decreased damage evidenced by lower caspase activity and increased FABP1 expression. From these data we conclude that while the use of NaHS constitutes a novel nongenetic strategy to protect cells and organs against hypothermia/rewarming injury, the induction of a serotonergic vesicular phenotype represents a novel genetic strategy to protect against this damage.

We demonstrate the successful induction of a vesicular, serotonergic phenotype in VTSMAC, as evidenced by an increase in the production and storage of serotonin in cells. We observed the presence of vesicles in VTSMAC very similar to those observed in DDT_1_. Increased levels of compounds such as collagen and serotonin was found in medium in response to hypothermia treatment both in DDT_1_ and VTSMAC, further substantiating that hypothermia triggers the secretion from cellular vesicles. Apart from the secretion of serotonin, our study also identifies hypothermia to cause secretion of collagen from VTSMAC and DDT_1_, which is related to the vesicular phenotype as there is limited secretion from non-transfected SMAC without vesicles.

Similarly to DDT_1_ cells, hypothermic treatment of VTSMAC induces the release of serotonin from the vesicular system. Apparently, the re-uptake of serotonin is a crucial step in governing protection, as the protection from hypothermic damage was abrogated by inhibition of serotonin reuptake following treatment of VTSMAC with a SSRI such as Fluoxetine. While serotonin itself has antioxidant properties [19], the protection of VTSMAC against hypothermia/rewarming damage is likely due to the production of H_2_S, as the protective effect was annihilated by the CBS inhibitor AOAA and CBS siRNA. The mechanism we previously identified [2] is based on serotonin preventing hypothermia/re-warming induced cell apoptosis by increasing H_2_S formation through CBS upregulation and probably allosteric activation. The upregulation of CBS seems however independent of 5-HT receptor activation, as serotonin effect was unaffected by the non-selective 5-HT receptor antagonist, ketanserine. [2]. The precise mechanism of CBS upregulation following serotonin reuptake remains to be elucidated. Thus, VTSMAC gain phenotypical characteristics and display resistance against hypothermic induced cell death similar to DDT_1_ cells. Our findings are in line with the protective effect of serotonin treatment in SMAC [2].

Our results demonstrate that the induction of a vesicular serotonergic phenotype also protects kidney tissue, as evidenced by increased cell survival following cold static storage. Likely, the mechanism of protection is similar to those of VTSMAC, in view of the increased expression of CBS and increased H_2_S levels in hypothermia preserved kidney tissue transfected with TPH-1 and VMAT-1. The protection from hypothermic kidney damage by serotonin is in line with data showing protection in transplanted kidney by the bioamine dopamine [20].

Increased production of H_2_S is likely to represent the leading mechanism in the protection of TPH-1 and VMAT-1 transfected cells and tissues against hypothermic damage. Hypothermia damage to cells has been hypothesized to be mainly due to a burst of reactive oxygen species (ROS) upon rewarming. Particularly during the rewarming phase, low ATP production, Ca^2+^ overload and cell swelling result in apoptotic cell death. The presence of oxidative damage is regarded the most important adverse event following hypothermia/rewarming [7], [21]. The exogenous administration of H_2_S, either as a gas or as a salt, has been found to limit cell death in different in vitro models from ROS associated cell damage, such as ischemia-reperfusion injury of myocardium [22], kidney tissue [23], epithelial cells [24] and liver cells [3]. Further, H_2_S has been shown to directly scavenge free radicals [25]. We previously showed increased production of endogenous H_2_S to protect cells from hypothermic damage and identified CBS as the main enzyme involved [2]. This mechanism is likely to convey the protective effect of TPH-1 and VMAT-1 expression both in cells and tissue, in view of its abrogation of by the CBS inhibitor AOAA. Involvement of CBS is the more likely, as the enzyme is downregulated by hypothermic storage as found by us and previously in rat kidney [26]. Our data show that expression of CBS is restored both in transfected cells and tissue.

While we demonstrate H_2_S production through CBS after the induction of a serotonergic vesicular phenotype in kidney to be a protective factor against hypothermia, others have implicated CSE (cystathionine gamma lyase) as the principal H_2_S producing enzyme in the kidney with protective properties against ischemia reperfusion damage [16]. In our experiments we found only CBS to be implicated in protection against hypothermia-rewarming induced damage, as demonstrated by the attenuation of protection by specific CBS siRNA knockdown and by treatment with AOAA. Further, we did not find a significant change in the expression of CSE following serotonin treatment (data not shown). Exploiting the beneficial effects of production and storage of serotonin and the consequent release and production of H_2_S through an internal source is possibly one of the most strategic manipulations to consider when protection against hypothermia induced ischemia reperfusion is concerned. There is, however, few or no relevant data on ischemia/reperfusion or hypothermic injury being suppressed through the induction/presence of a vesicular phenotype which stores a protective molecule such as serotonin, while having the characteristic to be triggered release due to hypothermia and finally to induce the production of endogenous H_2_S through CBS.

In summary, H_2_S protects against hypothermia damage and the transfection with TPH-1 and VMAT-1 induces production and storage of serotonin in cultured cells and renal tissue, which upon release and re-uptake conveys resistance to hypothermia via the induction of H_2_S production through CBS. This finding may serve to develop novel strategies to improve viability of transplantation organs based on the induction of a triggered release system in cells where vesicular entities store and release a protective factor such as serotonin. Further, it contributes to understanding the importance of the presence and the role of a vesicular phenotype in protection of cells against a damaging stress such as hypothermia.

## Supporting Information

Figure S1 **TPH-1 protein expression increases in SMAC and kidney slices after transfection with TPH-1 and VMAT-1 sequences (transfected cells/tissue is referred to as VTSMAC and VTkidney).** A) TPH-1 expression in VTSMAC increases two fold compared to SMAC, B) TPH-1 expression in VTkidney increases two fold compared to normal kidney. Data are mean ± SEM (n ≥3 per group). * different to normal cells, P<0.05.
(TIF)
