# Supplementary figures and images for: Induction of VMAT-1 and TPH-1 Expression Induces Vesicular Accumulation of Serotonin and Protects Cells and Tissue from Cooling/Rewarming Injury
Source: PLoS One. 2012 Jan 12;7(1):e30400. doi: 10.1371/journal.pone.0030400 (PMC3257274; doi:10.1371/journal.pone.0030400)

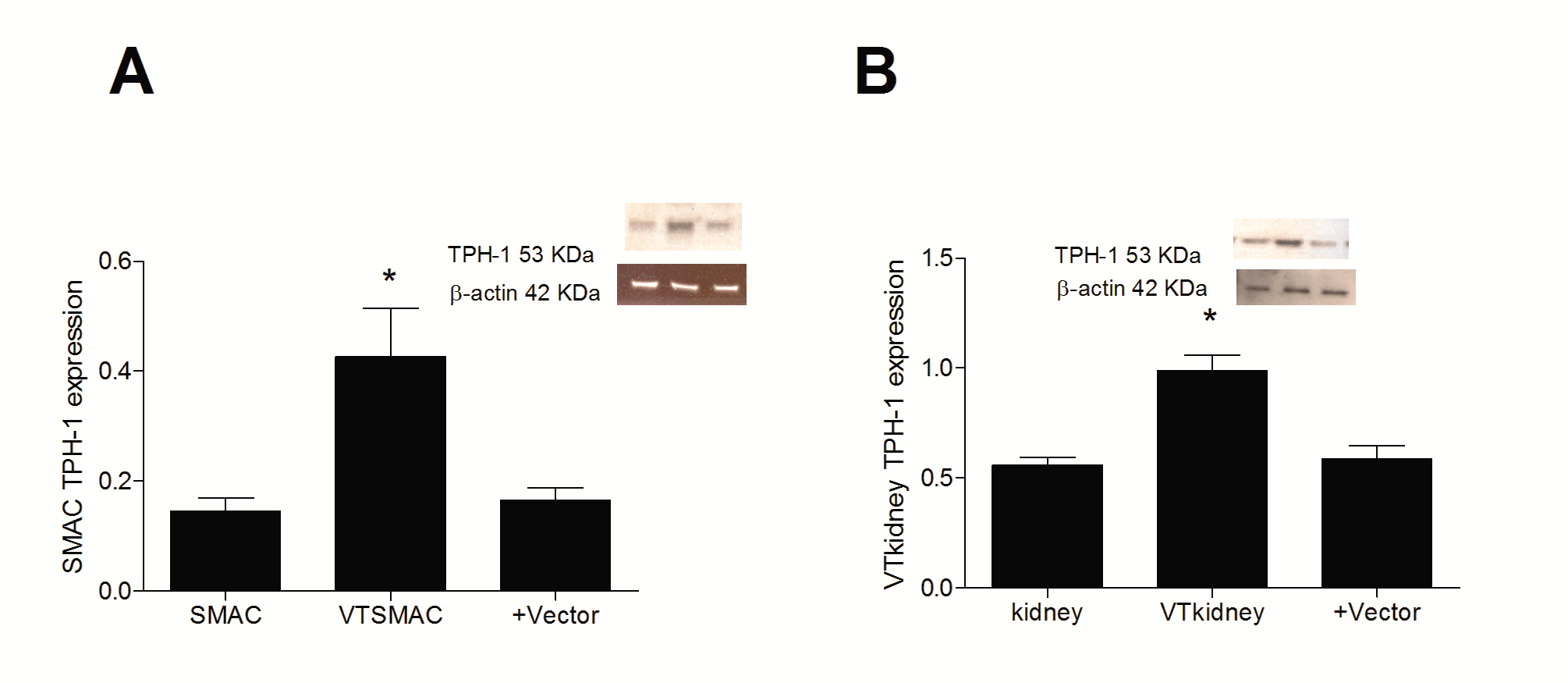

Supplement: Figure S1 — TPH-1 protein expression increases in SMAC and kidney slices after transfection with TPH-1 and VMAT-1 sequences (transfected cells/tissue is referred to as VTSMAC and VTkidney). A) TPH-1 expression in VTSMAC increases two fold compared to SMAC, B) TPH-1 expression in VTkidney increases two fold compared to normal kidney. Data are mean ± SEM (n ≥3 per group). * different to normal cells, P<0.05. (TIF) [file pone.0030400.s001.tif]
